# Supplementary material for: Differential Responders to a Mixed Meal Tolerance Test Associated with Type 2 Diabetes Risk Factors and Gut Microbiota—Data from the MEDGI-Carb Randomized Controlled Trial
Source: Nutrients. 2023 Oct 15;15(20):4369. doi: 10.3390/nu15204369 (PMC10609681; doi:10.3390/nu15204369)
Supplement: Supplementary file 1 [file nutrients-15-04369-s001.zip › nutrients-2622916-supplementary.pdf]

nutrients

MDPI

Supplementary data

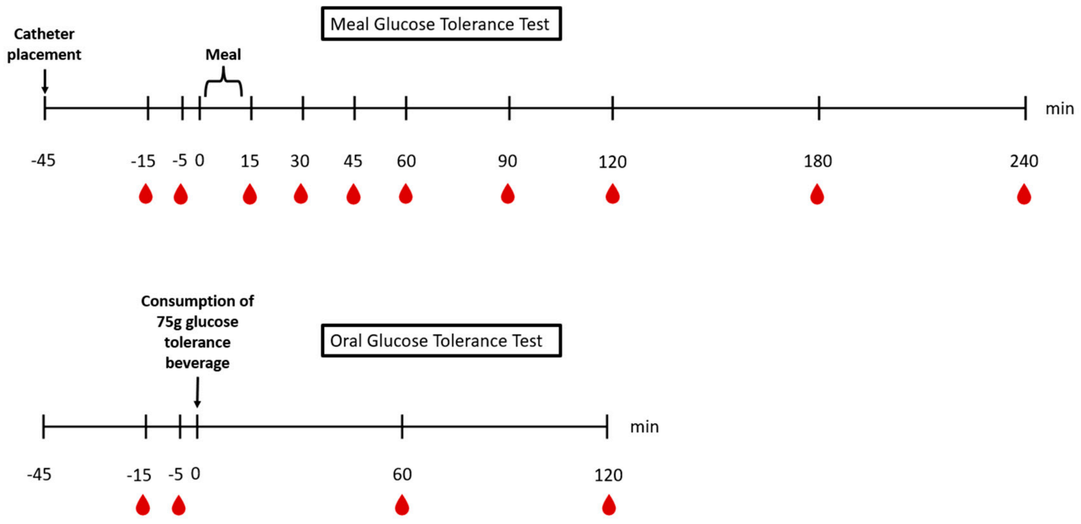

Figure S1: Meal- and Oral Glucose Tolerance Tests overview. Blood drops indicate the time point in minutes when blood samples were taken.

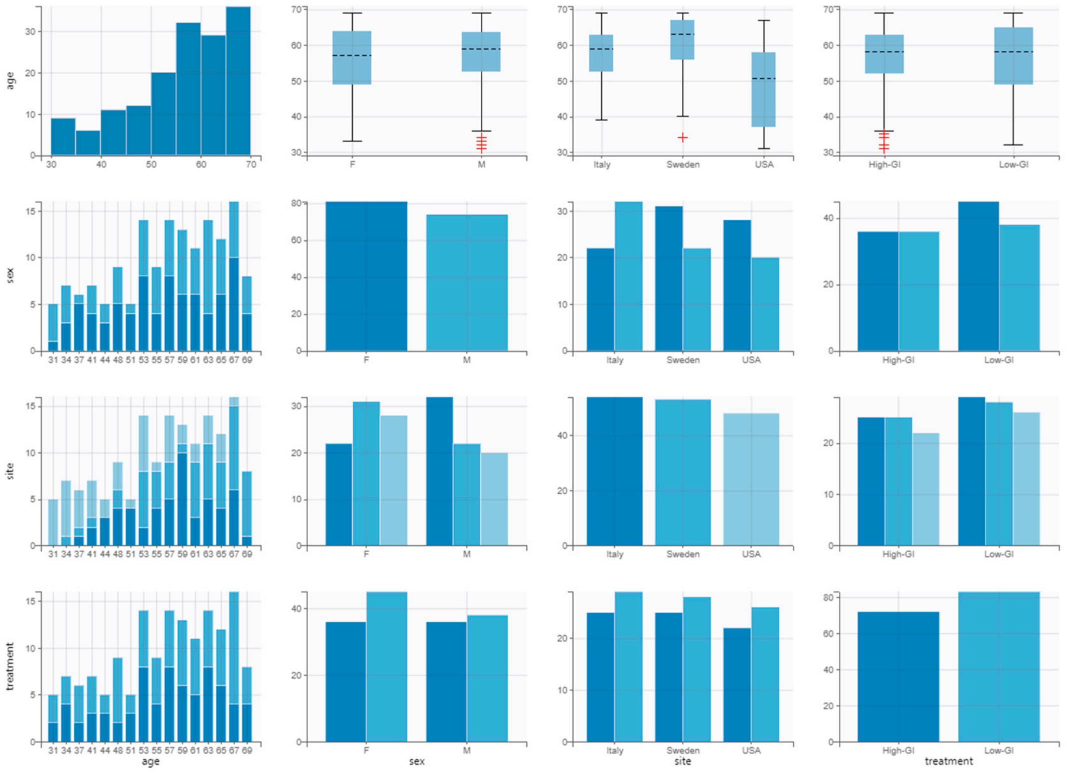

Figure S2: Covariate analysis at baseline. Each plot represents the joint analysis of pairwise covariates, e.g., the first row of the second column represents the distribution of age in men and women. Suspected outliers are plotted with red plus signs in box plots.

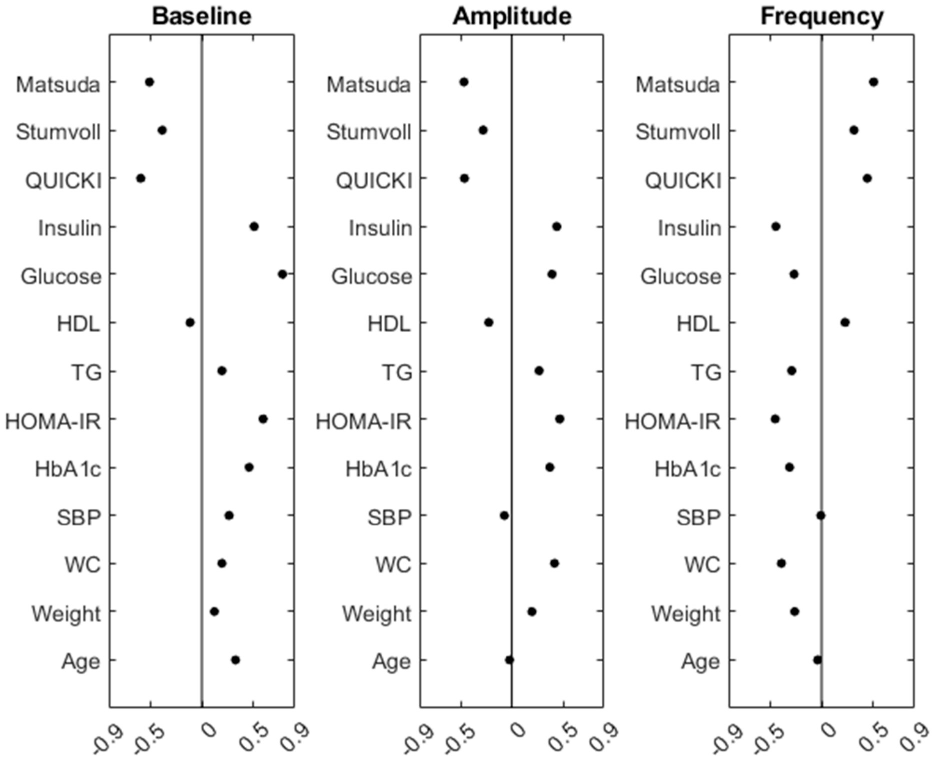

Figure S3: Spearman correlation between T2DM risk markers and estimated parameters from the model using baseline data.

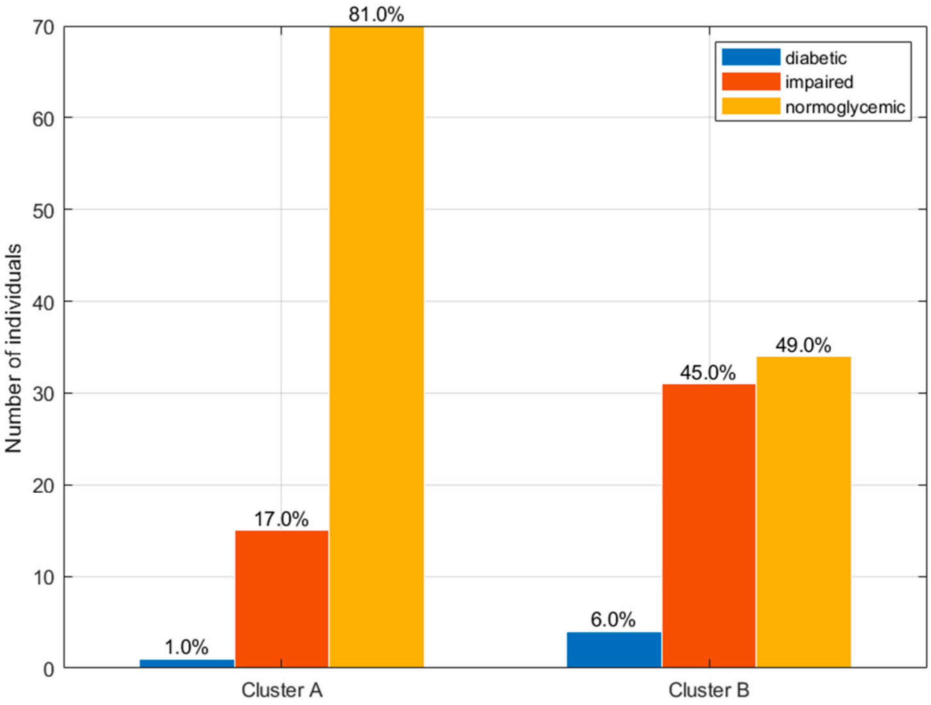

Figure S4: Distribution of glycemic control classification in the two identified response clusters.

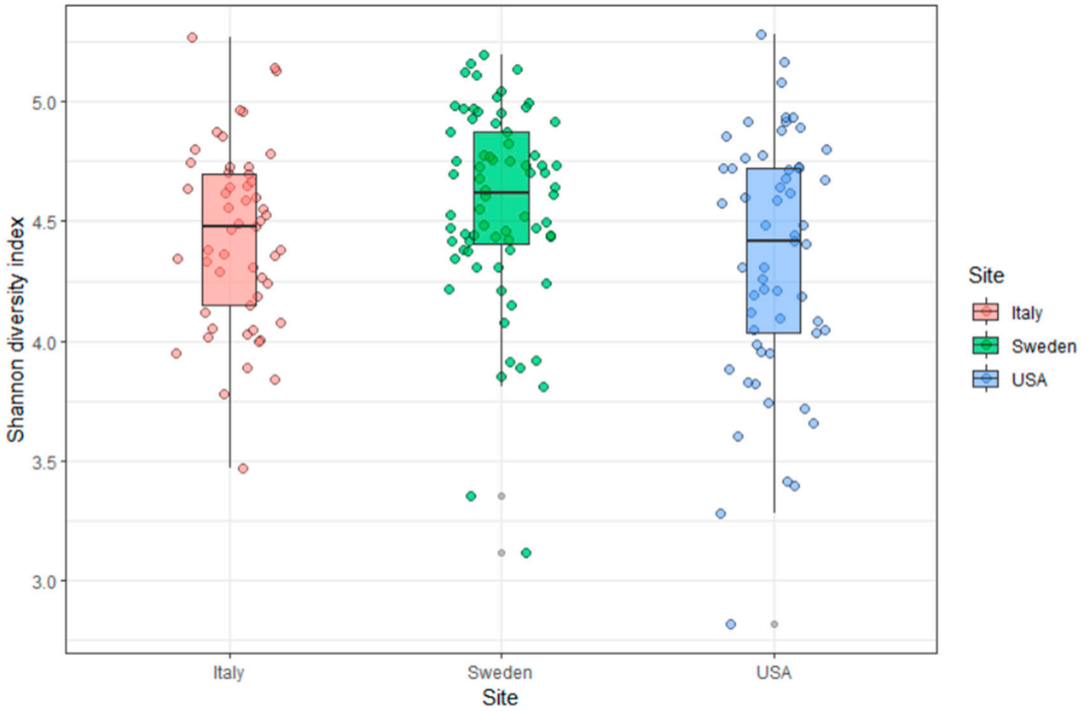

Figure S5: Shannon diversity index of the microbiota genera shown per site using boxplots.
